# Gender Identity and Gender Role in DSD Patients Raised as Females: A Preliminary Outcome Study

**DOI:** 10.3389/fendo.2013.00086

**Published:** 2013-07-15

**Authors:** Oya Ercan, Seyhan Kutlug, Omer Uysal, Mujgan Alikasifoglu, Derya Inceoglu

**Affiliations:** ^1^Pediatric Endocrinology and Adolescent Divisions, Pediatrics Department, Cerrahpasa Medical Faculty, Istanbul University, Istanbul, Turkey; ^2^Pediatrics Department, Cerrahpasa Medical Faculty, Istanbul University, Istanbul, Turkey; ^3^Department of Biostatistics and Medical Informatics, School of Medicine, Bezmialem Vakif University, Istanbul, Turkey; ^4^Adolescent Division, Pediatrics Department, Cerrahpasa Medical Faculty, Istanbul University, Istanbul, Turkey; ^5^Academic Success Monitoring and Counseling Program, Center for Individual and Academic Development-CIAD, Sabanci University, Istanbul, Turkey

**Keywords:** gender role in DSD, gender identity in DSD, outcome in DSD, females with DSD, psychosexual development in DSD

## Abstract

Gender identity and gender role are expected to be consistent with gender assignment for optimal DSD management outcome. To our knowledge, our study is the first to attempt evaluation of gender related outcomes in Turkish DSD patients. After receiving institutional ethical board approval and subject (or parent) informed consent, subjects with DSD raised as girls (22 patients 46 XX DSD, 11 patients 46 XY DSD) answered 566 questions of the Minnesota Multiphasic Personality Inventory (MMPI) questionnaire including 60-item Masculinity-Femininity (MF) subscale which was the focus in this study. Controls (*n*: 50) were females similar to the probands in age, level of education, relationship status, and having a job or not also answered all questions. The answers were evaluated by a trained psychologist (Derya Inceoglu) on MMPI. For statistical purposes, seven findings were obtained from the data related to the MF subscale from the patients and controls. Of these seven findings (S1–S7), two were associated with masculinity (S3–S4) and another two were associated with femininity (S5–S6). In DSD patients, the percentages of masculinity findings were significantly higher when compared to controls (*p* < 0.001 and *p* < 0.001 for S3 and S4, respectively). In controls, the percentages of femininity findings were significantly higher when compared to DSD females (*p* < 0.001 and *p* < 0.001 for S5 and S6 respectively). There was no significant difference between 46 XX DSD patients and 46 XY DSD patients with respect to the percentage of any of the seven findings. Two patients requested gender change to male; only these two patients had the finding stating that sexual impulses could come to existence as actions (S7). In conclusion efforts to identify modifiable factors with negative impact and thus modifying them, and professional guidance may be important in minimizing the encountered gender related problems in DSD patients.

## Introduction

Although previous studies indicated contentment with the sex of rearing in the majority of patients with disorders of sex development (DSD) raised as females (1–3), more recent reports suggest revision of views on female gender assignment in DSD patients to minimize unfavorable outcomes (4–12).

At least three aspects of human psychosexual differentiation show sex-dimorphic differences which are gender identity, gender role, and sexual orientation (13). Previously in Turkish children with DSD raised as females aged 6 months to 14 years at the time of evaluation, gender role was found to be appropriate for their gender of rearing but a male or neutral gender self-perception was reported in some of the girls (14). In another study from Turkey, the results of surgical modalities for 55 children with ambiguous genitalia were evaluated after an average period of 4.1 years after the surgeries (15). Some of these patients are some of the subjects of the present study. In that study, among the 10 patients of postpubertal age at that time, none of them had sexual experience and issues related to gender role and gender identity had not been evaluated.

Thus, in this study, we aimed to evaluate the two gender related long-term outcomes, namely gender identity and gender role, in our DSD patients raised as females. To our knowledge, our study is the first to attempt evaluation of gender related outcomes in Turkish DSD patients of postpubertal age.

## Materials and Methods

Seventy-five potential probands were identified from the registry of DSD patients evaluated by the multidisciplinary team of DSD in Cerrahpasa Medical Faculty of Istanbul University. These individuals were those who were raised as females and live as females, and were above 16 years of age at the time of investigation with both 46 XX and 46 XY karyotypes. From these, 42 probands could be contacted (8 declined to participate) and 34 probands accepted to participate.

Potential controls were chosen from the same aged females as the patients, initially two to three people for each patient. They were females who were the sisters or the friends of the patients, or they were chosen from the people where the authors work or live. After age, those controls at the same level of education and at the same relationship status with the probands were chosen respectively. Finally having a job or not was taken into consideration with a final number of controls being reduced to 50. The 50 controls were finally similar to the probands in age (mean ± SD: 22.53 ± 5.11 years), level of education, relationship status, and having a job or not. Relationship status of the patients and controls is shown in Table 1.

**Table 1:** **Relationship status of the patients and controls**.

	Married	Single	Divorced	Boyfriend (+)
Patients (*n*: 34)	2	30	2	12
Controls (*n*: 50)	3	44	3	18

As our aim was to get an overview of how our patients were with respect to gender role and gender identity in this study, only diagnoses, height, BMI, and number of operations were mentioned as clinical features. For evaluation of gender identity and gender role the instrument used was the Minnesota Multiphasic Personality Inventory (MMPI) which contains 3 validity scales and 10 clinical scales. This inventory was developed by SR Hathaway and JC Mc Kinley (16). It was validated in the Turkish population by Isik Savasir (17).

The focus in the context of this study was the 60-item masculinity-Femininity (MF) subscale of the MMPI. However, both patients and controls answered the 566 questions of the inventory. The questions had to be answered as “true” and “false” or left blank if could not be answered. Answering needed to be completed on the same day on one occasion. For the present study, only the results related to the MF-subscale were analyzed in the context of the whole questionnaire.

The answers were evaluated by one of the authors who is a trained psychologist on MMPI (Derya Inceoglu). The profiles of both patients and controls were determined after they were drawn on specific profile papers for MMPI. The evaluation of the MF subscale could then be made in the context of the profiles and interpreted.

Approval of the ethical board of medical, surgery, and drug investigations of Cerrahpasa Medical Faculty of Istanbul University for the study was obtained. Written informed consent from the parents and adult individuals (>18 years) and assent of those 16–18 years were obtained.

### Statistical analysis

For comparisons between controls and patients, and between 46 XY DSD and 46 XX DSD Chi square and Fisher test were used. Mann–Whitney test was used only to compare sibling numbers. To compare the means for age *t*-test was used. Significancy level was<0.05.

## Results

One questionnaire was not considered valid; thus results of 33 patients were included in the analyses. The ages of the 33 patients ranged from 16 to 32.6 (mean ± SD: 22.53 ± 3.38) years. Clinical diagnosis and DSD type of these patients are shown in Table 2. All the patients except one (late diagnosis 5-alfa reductase deficiency) had one to three operations.

**Table 2:** **Clinical diagnosis and DSD type of patients**.

DSD type	Diagnosis	Age (years)
46 XX DSD (*n*: 22)	CAH-sv (*n*: 15)	23.38 ± 5.07
	CAH-sw (*n*: 6)	
	P. Arom def (*n*: 1)	
46 XY DSD (*n*: 11)	AIS (*n*: 6)	20.85 ± 4.97
	5α red. def (*n*: 4)	
	Ovotest. DSD (*n*: 1)	

Between patients and controls and, between 46 XY DSD and 46 XX DSD there was no significant difference with respect to age, having a boyfriend, level of education, having a job, and number of siblings. Marital status could not be compared statistically. Height was 150.45 ± 7.91 cm in 46 XX DSD and 171.45 ± 7.93 cm in 46 XY DSD which was statistically significant (*p*:0.000). BMI was similar in the two groups.

From the interpretations of the MF-subscale data from the patients and controls, outstanding findings were grouped into seven for statistical purposes. Of these seven findings (S1–S7), two were associated with masculinity (S3–S4) and another two were associated with femininity (S5–S6). These findings were as follows:

S1: gender identity is not properly developed,
S2: gender identity in between,
S3: behaviors are appropriate for male gender identity,
S4: emulation of male gender behaviors and copying of them from time to time/not embracing female gender identity,
S5: behaviors are appropriate for female gender roles,
S6: embracement of female gender identity/gender identity compatible with female gender identity,
S7: sexual impulses can come to existence as actions.

As shown in Table 3, in patients, the percentages of S3 and S4 were significantly higher than in controls (*p* < 0.001 and *p* < 0.001 respectively). There was no significant difference between 46 XY DSD and 46 XX DSD patients from this respect (Table 4).

**Table 3:** **Comparison of the percentages of the findings between patients and controls**.

Finding	Controls (*n* = 50)		DSD (*n* = 33)		*p*
	*n*	%	*n*	%	
S1	17	34	13	39.3	0.617
S2	2	4	6	18.1	0.054
S3	0	0	15	45.45	<0.001
S4	0	0	11	33.3	<0.001
S5	45	90	10	30.3	<0.001
S6	46	92	14	42.42	<0.001
S7	0	0	2	6.06	0.155

**Table 4:** **Comparison of the percentages of the findings between 46 XX DSD and 46 XY DSD**.

Finding	46 XX DSD *n* = 22		46 XY DSD *n* = 11		*p*
	*n*	%	*n*	%	
S1	9	40.9	4	36.4	1
S2	5	22.7	1	9.1	0.637
S3	9	40.9	6	54.5	0.458
S4	6	27.3	5	45.5	0.437
S5	9	40.9	1	9.1	0.109
S6	11	50	3	27.3	0.278
S7	1	4.5	1	9.1	1

Again, as shown in Table 3, the percentages of the findings S5 and S6 were significantly lower in patients than in controls (*p* < 0.001 and *p* < 0.001, respectively). There was no significant difference between 46 XY DSD and 46 XX DSD patients from this respect also (Table 4).

One 46 XX DSD patient and especially her family expressed desire for changing to the male sex. She had quitted treatment. Her findings were S2, S3, S4, and S7. One 46 XY DSD patient expressed desire for changing to the male sex. Her findings were S3, S4, and S7.

## Discussion

Of the seven findings which were obtained from our data, two findings (S1, S2) were related to undeveloped gender identity, or to gender identity not differentiated to one direction, neither to female nor to male. These two findings were similarly present in both patients and controls: the presence of these two findings in the patients seems to be a normal element of gender development at least in our community. However, the percentages of the two other findings (S3, S4) which were related to male gender were significantly higher in the DSD patients than in controls. In fact, in none of the controls these findings were present which indicates that these findings are related to DSD.

One of these findings state that behaviors are appropriate for male gender identity. The DSD group in our study consists of both 46 XY individuals with androgen secreting testicular tissue and, 46 XX individuals who, except one, have congenital adrenal hyperplasia. Thus, individuals in both groups might have had more androgen exposure than that in normal females at least during fetal life. Androgens have been suggested to exert an organizational effect on higher brain function during fetal development (18).

Female adolescent and adult CAH patients have been studied thoroughly from this respect and have been found to show masculinized gender behavior (13, 19–22) as we have also found in our patients, or recall cross-gender role behavior (13, 23). Congenital adrenal hyperplasia patients constitute about 2/3 of our DSD patients and all except one of the 46 XX patients in our study. Besides, no statistical difference was found between 46 XY DSD and 46 XX DSD groups with respect to the percentage of the aforementioned finding related to masculinized behavior. Thus, this finding could be discussed in the context of the reported behavioral masculinization in females with CAH.

It is well known that girls with CAH show more male-typical behavior and less female-typical behavior than other girls (24). These have been mostly attributed to prenatal androgenic influences on the developing brain. However, explanation for these findings from the social-cognitive perspectives has also been the subject of interest (24). The unexpected finding of Mattila et al. (12) which stated that CAIS patients were more androgynous than both female and male controls and also more androgynous than PAIS patients might further point to the complexity of gender related issues in DSD patients. Interestingly, in the aforementioned study there was no difference between the whole groups of patients with 46 XX DSD and 46 XY DSD from the respect of androgyny as we have not found any difference between the two groups with respect to masculinized behavior.

The second finding related to male gender which was significantly higher in our patients than in controls was emulation of male gender behaviors and copying of them from time to time or not embracing female gender identity. About 33% of the DSD patients had this finding while none of the controls had it. This might be interpreted as one third of our DSD patients would like to behave like males and that they would have felt more comfortable if they had lived as males. Again, no statistical difference was found between 46 XY DSD and 46 XX DSD groups with respect to the percentages of this finding.

Satisfaction with the sex of rearing has been investigated previously in women with DSD (1, 3, 13, 24–27).

In CAIS, despite previous studies which reported satisfaction with having been raised as females and no desire for gender reassignment (25, 26), two recent reports suggested one should give more than a second thought before assigning female gender to patients with CAIS (6, 12). Migeon et al. (3) reported only 22% dissatisfaction with the sex of rearing in a group of 46 XY individuals born with perineoscrotal hypospadias and who were assigned to the female gender. These authors concluded in their study that either male or female sex of rearing could lead to successful long-term outcome for the majority of cases of severe genital ambiguity in 46 XY individuals (3). On the other hand, the finding of emulation of male gender behaviors and copying of them from time to time or not embracing female gender identity was found in almost half (45.5%) of our 46 XY DSD patients (all born with genital ambiguity except one CAIS patient) possibly indicating to a gender identity discomfort in these individuals. What makes our finding strong is that it was not found in any of the controls. Whether this would lead to gender dysphoria in time or remain only as discomfort is an unknown for the time being. In the study by Mattila et al. (12), in 46 XY DSD patients a somewhat more conflicted gender identity than those with the 46 XX karyotype was reported. As we did not find a significant difference between 46 XY and 46 XX individuals from this respect, one poses the question of whether the difference is due to cultural aspects.

Cohen-Kettenis (4) in her overview stated that patient-initiated gender reassignment in a subgroup of 46 XY individuals who were raised as girls occurred frequently in adolescence and early adulthood as it has also been the case in one of our 46 XY DSD patients. This patient who was at her early adulthood at the time of the study had bilateral orchiectomy beyond infancy, tried to live as a female, fell in love with a female, and finally took this study as an opportunity to express that she wanted to live as a male.

Hines et al. (23) assessed core gender identity in CAH in individuals between the ages of 18 and 44 years for both lifetime and for the most recent 12 months. The items targeted gender identity at each time period asked whether the participant enjoyed being a person of her own sex, wished to be a person of the other sex, or thought that she was psychologically a person of the other sex. It was shown that women with CAH were less satisfied with the female sex assignment than controls and that there was a significant correlation between the recalled male-typical play in childhood and reduced satisfaction with the female gender over the lifetime but not over the past 12 months, albeit the correlation of moderate size, which suggested that this dissatisfaction may be reduced in adulthood. However, the difference in gender identity between females who do and do not have CAH was found to be only about 11% of the size of the sex difference which was much smaller than the difference in childhood gender role behavior (24). Hines (24) interpreted this finding as the possible sign of more dramatic effects of CAH on childhood gender role behavior than on gender identity in adulthood. Long et al. (28) found that women with CAH showed a decrease in masculinity across developmental stages, such that by adulthood, there were no significant differences in masculinity between controls and the women with CAH. In our study, however, emulation of male gender behaviors and copying of them from time to time or not embracing female gender identity was found in 27.3% of individuals in our 46 XX DSD group (21/22 CAH) at a mean age of 23.38 years and was significantly higher than in controls while behaviors were appropriate for male gender identity in 40.9% of these patients. Thus while this might mean that the female identification in about one third of our 46 XX DSD women may not be as strong or secure as it is in women without DSD as also previously mentioned (24), it might also suggest that male-typical behavior attributed to patients with CAH in childhood not only exists in childhood (24) but also in young adulthood and again in more than those individuals who might not have a strong or secure female identification.

Dessens et al. (1), in their review found that the large majority of the patients with CAH raised female did not feel gender dysphoric. However, 5.2% of the patients had serious problems with their gender identity. Nevertheless these authors concluded that gender identity development, at least in patients with classical CAH, was remarkably flexible as it had been previous reported and that assignment to the female gender as a general policy for 46 XX patients with CAH appeared justified (1, 24). On the other hand, more recently Lee et al. (9) and Houk and Lee (8) proposed consideration of male assignment for 46, XX patients who fully developed male genitalia based on available outcome data. One of our CAH patients with salt-losing CAH who together with her family also took this study as an opportunity to protest being a female provides an additional datum. This patient was dressed as a male and behaved as a male. She had severe and frequent attacks of salt-loss in early childhood (maybe due to non-compliance although never confessed and inconsistent or absent glucocorticoid treatment more recently).

The two other findings (S5, S6) were those related to female gender. These two findings were significantly lower in the patients than in controls. Similarly, Warne et al. (2) reported lower femininity scores in intersex females than in control females in a group of both 46 XX DSD and 46 XY DSD patients, a group of patients similar to ours. On the other hand, as findings related to male gender were significantly higher in our patients than in controls, lower percentages of findings related to female gender in our patients were expected. This shows the consistency of our results. Again there was no significant difference between 46 XX DSD and 46 XY DSD with respect to these two findings (S5, S6) also. However, behaviors seemed to be less frequently appropriate for female gender roles in the 46 XY DSD group than those with 46 XX DSD (9.1 vs. 40.9%) albeit not significantly. Further investigation is needed to understand whether a meaningful difference exists between 46 XX DSD and 46 XY DSD from this respect. On the other hand, in a recent study, assessment of gender role orientation revealed no differences between the femininity indices of the 46 XX and 46 XY patients. However, their 46 XY DSD group was comprised of only AIS patients (12). Interestingly, Pappas et al. (29) reported that individuals with 46 XY DSD raised female reported more femininity than those raised male due to an increase in feminization during adolescence and adulthood. These authors concluded that their results are consistent with the idea that socialization/learning contributes to gender role development in humans in addition to data from others demonstrating endocrine influences. Long et al. (28) found that self-reported femininity decreased in a dose-response manner in adult women with CAH due to 21-hydroxylase deficiency, according to prenatal androgen exposure. This might also hold true for 46 XY DSD and thus set the balance between endocrine influences and socialization/learning depending on the specific types of 46, XY DSD present in the group investigated in this study. From this respect, studies performed in diagnostically homogenous groups of 46 XY DSD, thus putting an effort to equalize prenatal androgen exposure, might be useful.

Long et al. (28) reported that in adult women with CAH that femininity increased through childhood, adolescence, and adulthood. However, overall, femininity was greatest for control women while women with salt-losing CAH remained significantly less feminine than controls into adulthood. In our 46 XX DSD group (21/22 CAH) at a mean age of 23.38 years, our two (S5, S6) female gender related findings were present in only 40.9 and 50.0% of the patients.

Lastly, in 6.06% (2/33) of our patients, we found that sexual impulses could come to existence as actions while in none of the controls this finding was present. It might be worthy to discuss this finding as this finding *per se* was not reported in DSD patients before although the difference between the patients and controls was not significant. The finding we mention here was present in one 46 XX DSD and in one 46 XY DSD patient. Interestingly, they were the two patients who requested gender change. Previously, it was stated that females with CAH and females who are exposed to progestins prenatally show increased propensities to aggression, at least as indicated by responses to interviews and questionnaires. However, it was also stated that whether these increased propensities would translate into aggressive behavior was not clear (24). It was also stated that salt-wasting CAH patients showed a higher level of activity (20). Although we do not know the extent of the commonality of our finding with the findings stated above, we might define our finding as doing positive or negative things in aggression and ambitiously due to sexual impulses. Whether the presence of this finding in female DSD patients might be an indicator of the wish for gender change or only apparently so in our patients needs further investigation.

The differences from previous literature we observed with respect to satisfaction with the sex of rearing (3) and the security of female identification (28) might somewhat be related to methodological differences: Migeon et al. (3) made use of semi-structured interviews besides a written questionnaire. These authors also investigated sexual functioning which was not the subject of our study. In the study by Long et al. (28) only one question was asked. In our study however we have used a questionnaire with 566 questions, 3 validity scales, and 10 clinical scales and the data related to the MF subscale was interpreted in the context of the whole questionnaire.

Minnesota Multiphasic Personality Inventory was chosen as the instrument for this study because at the time of the measurement (as it is also so now), to our knowledge, this was the only measure which was adapted to Turkish and validated in Turkish people we had at hand to measure gender identity and gender role in patients of postpubertal age. The fact that it is an old version might raise a question about its usefulness. Although MMPI was started being used in the 1950s, in 1988, it was shown that the behavioral correlates established a generation ago were still valid at least for college students who were probably at the same age with our patients and focusing of the study, i.e., 60-item MF subscale was the same with ours (30). After this validation, another generation has passed but considering it was valid after one generation, one can consider it might not be much different after another generation especially in a relatively traditional country as ours. Besides, the same version of the questionnaire was used in French, Canadian, and Italian individuals in the 2000s (31–33). In the French study, results were homogenous in respect of Rorschach card III and MMPI MF scale which were both accepted to be linked to gender identification (31).

One might also argue that a written questionnaire but not other techniques were used in our study. In the previously published studies we have cited in this study, only in some, semi-structured interview/interview with or without a written questionnaire (3, 13, 19, 20, 26) was used but in others only a written questionnaire and an inventory (12, 22), a questionnaire (23), or one question (28) were made use of. Zucker (34) describes one of the measures (28) as not having been subjected to formal psychometric analysis and describes two others (13, 23) as the psychometrics of which have not been studied to a great extent and suggests the use of a new measure which was designed to assess gender identity and gender dysphoria in a more dimensional manner but this measure was neither adapted to Turkish nor validated in Turkish people. Moreover, the questionnaire we used in this study, the MMPI, is a measure the psychometric analysis of which has been studied.

One of the limitations of our study is that the results were obtained from the participants; non-participants and those potential probands who could not be contacted might have different characteristics than those who could be contacted. Another limitation is that the results are relevant to the time of the study; there might be important changes in the characteristics evaluated as the patients get older. Third limitation is that the analysis was confined to the results related to the MF subscale of the instrument; psychosocial findings were not analyzed in this study. Last but not the least limitation might be the cultural effect: in our country, male domination and lack of capacity for fertility might be potential and important issues affecting gender role and gender identity in females with DSD.

In conclusion, our study results suggest that besides the presence of male gender behaviors and less behaviors appropriate for female gender roles in a half and in one third of our female raised DSD patients respectively, despite absence of cross-gender identity development in most of our patients, in almost half of those with the 46 XY karyotype and in a third of those with the 46 XX karyotype a gender identity discomfort exists.

Such a discomfort might result in unhappiness because by contrast to the disclosure of the wish for gender change, it remains hidden and might result in desperation. Thus, besides efforts identifying the modifiable factors with negative impact and, careful medical treatment and follow-up, regular psychological support through all developmental stages and gender counseling starting from young adulthood should be a routine part of management to minimize the encountered gender related problems in DSD patients.

## Conflict of Interest Statement

The authors declare that the research was conducted in the absence of any commercial or financial relationships that could be construed as a potential conflict of interest.
